# Anemoside B4 alleviates arthritis pain via suppressing ferroptosis‐mediated inflammation

**DOI:** 10.1111/jcmm.18136

**Published:** 2024-02-09

**Authors:** Chenlu Guo, Yuanfen Yue, Bojun Wang, Shaohui Chen, Dai Li, Fangshou Zhen, Ling Liu, Haili Zhu, Min Xie

**Affiliations:** ^1^ School of Pharmacy Hubei University of Science and Technology Xianning China; ^2^ Department of Obstetrics Xianning Central Hospital, First Affiliated Hospital of Hubei University of Science and Technology Xianning China; ^3^ Hubei Key Laboratory of Diabetes and Angiopathy, School of Basic Medical Sciences, Xianning Medical College Hubei University of Science and Technology Xianning China; ^4^ Department of Pharmacy Matang Hospital of Traditional Chinese Medicine Xianning China

**Keywords:** anemoside B4, arthritis pain, ferroptosis, glycogen synthase kinase 3β, neuroinflammation

## Abstract

Chronic pain is the key manifestations of rheumatoid arthritis. Neuroinflammation in the spinal cord drives central sensitization and chronic pain. Ferroptosis has potentially important roles in the occurrence of neuroinflammation and chronic pain. In the current study, mouse model of collagen‐induced arthritis was established by intradermal injection of type II collagen in complete Freund's adjuvant (CFA) solution. CFA inducement resulted in swollen paw and ankle, mechanical and spontaneous pain, and impaired motor coordination. The spinal inflammation was triggered, astrocytes were activated, and increased NLRP3‐mediated inflammatory signal was found in CFA spinal cord. Oxidative stress and ferroptosis in the spinal cord were manifested. Meanwhile, enhancive spinal GSK‐3β activity and abnormal phosphorylated Drp1 were observed. To investigate the potential therapeutic options for arthritic pain, mice were intraperitoneally injected with AB4 for three consecutive days. AB4 treatment reduced pain sensitivity and increased the motor coordination. In the spinal cord, AB4 treatment inhibited NLRP3 inflammasome‐mediated inflammatory response, increased antioxidation, decreased mitochondrial reactive oxygen species and ferroptosis. Furthermore, AB4 decreased GSK‐3β activity by binding with GSK‐3β through five electrovalent bonds. Our findings indicated that AB treatment relieves arthritis pain by inhibiting GSK‐3β activation, increasing antioxidant capability, reducing Drp1‐mediated mitochondrial dysfunction and suppressing neuroinflammation.

## 1 INTRODUCTION

Rheumatoid arthritis (RA) is a common systemic inflammatory autoimmune disease, and affects approximately 0.5%–1% of the population worldwide.^1^ Chronic pain is the key manifestations of RA, which impairs patients' physical function.^2^ Nonsteroidal anti‐inflammatory drugs, disease‐modifying antirheumatic drugs and glucocorticoids are used to treat pain of RA.^3^ Research on the mechanisms of RA pain is helpful for therapy of pain management.

Central sensitization, the key mechanism of chronic pain, plays a pivotal role in RA pain.^4^ According to the central sensitization inventory, about 41% of RA patients have central sensitization syndrome.^5^ During RA processing, destruction and disability of joint evoked pain‐producing agents, activated the primary afferents, stimulated nociceptive signals and inflammatory response in the spinal cord and supraspinal, caused central sensitization and pathological pain.^2^ Neuroinflammation in the spinal cord is a major component in facilitating neuronal excitability and maintaining of chronic pain.^6^ Neuroinflammation is characterized as glial cells activation and inflammatory mediators release.^7^ In RA pain, spinal astrocytes are activated, undergo hyperplasia and hypertrophy, and release numerous pro‐inflammatory mediators such as interleukin (IL)‐1β, which are known to directly sensitize nociceptors and mediate pain.^8^ NLRP3 inflammasome is a crucial signalling node that controls the maturation of IL‐1 family cytokines.^9^ NLRP3 activation leads to inflammasome recruitment and results in the pro‐caspase‐1 activation and cleavage.^10^ Inflammatory caspase‐1 elicits the maturation and release of IL‐1β, and initiate pyroptosis.^10^, ^11^ In a mouse model of chronic neuropathic pain, divanillyl sulfone inhibits expression of NLRP3, reduces the production of mature caspase‐1 and IL‐1β, and increases the mechanical withdrawal threshold.^12^ In cancer‐induced bone pain model, NLRP3 inflammasome inhibitor MCC950 treatment restores the expression of NLRP3 inflammasome and significantly suppresses the upregulation of IL‐1β, attenuates mechanical allodynia.^13^ Thus, targeting against NLRP3 inflammasome activation is a potential therapeutics for chronic pain.

Mitochondria are the powerhouses of eukaryotic cells via oxidative phosphorylation, perform crucial functions in bioenergetics, metabolism and signalling, and associate with numerous diseases, such as Alzheimer's disease and Parkinson's disease.^14^, ^15^, ^16^ Our previous study on cancer‐induced bone pain animal showed morphologic and proteomic changes in mitochondria.^17^ Mitochondrial dysfunction is strongly correlated with inflammatory response. The highly oxidative environment causes mitochondrial membrane potential loss and consequently NLRP3 inflammasome activation and inflammatory cytokine secretion.^18^ Mitochondria is the main source of cellular reactive oxygen species (ROS). The complex I and III inhibitors treatment results in the loss of mitochondrial membrane potential and robust ROS production.^19^ Mitochondrial ROS released from dysfunctional mitochondria triggers NLRP3 inflammasome activation, which leads to the cleavage and release of proinflammatory IL‐1β. Mitochondria are involved in the painful peripheral neuropathies evoked by chemotherapy, diabetes and HIV.^20^ Moreover, NLRP3 inflammasome has been implicated in the pathology of painful diseases, including neuropathic pain, RA and chemotherapy‐induced peripheral neuropathy.^21^ Taken together, mitochondrial dysfunction and NLRP3 inflammasome activation appear to be contributed to the pathology of chronic pain.

Anemoside B4 (AB4), the bioactive ingredient of triterpenoid saponins in the traditional Chinese medicine pulsatilla, has the anti‐inflammation and analgesia effects.^22^ In mice model of ulcerative colitis, AB4 significantly reduces levels of proinflammatory cytokines IL‐1β, IL‐6 and TNF‐α.^23^ In acute lung injury model, AB4 blocks NLRP3 inflammasome activation and has a protective effect on lung injury.^24^ Until now, targeting protein and the underlying mechanism for AB4 on pain is still unclear. In our study, AB4 was intraperitoneally administrated on a mouse model of arthritis pain. The effects of AB4 on behaviours, spinal morphology and protein expression were analysed to clarify the analgesia mechanism of AB4 on arthritic pain. The study was helpful to explore and develop AB4 as an analgesic drug for arthritis pain.

## 2 MATERIALS AND METHODS

### 2.1 Antibodies and reagents

Anti‐IL‐1β (AF5103), GFAP (DF6040), caspase‐1 (AF5418), cleaved caspase‐1 (AF4022), NDUFB11 (F12300), DRP1 (DF7037), phospho‐DRP1 (Ser616) (AF8470), phospho‐DRP1 (Ser637) (DF2980), phospho‐GSK‐3β (Tyr216) (AF3335), GSK‐3β (BF0695) and β‐actin (T0022) antibodies were obtained from Affinity Biosciences. Anti‐NLRP3 (A12694), Nrf2 (A0674), GPX4 (A1933), DHODH (A6899) and cytochrome C (A13430) antibodies were purchased from ABclonal Technology. AB4 (B20060) and CFA (P2036) were purchased from Shanghai yuanye Bio‐Technology and Beyotime Biotechnology, respectively. HRP Goat anti‐rabbit IgG (H + L) (AS014) and HRP Goat anti‐mouse IgG (H + L) (AS003) used for western blotting were from ABclonal Technology. Goat anti‐rabbit IgG H&L (FITC) (ab6717) and Goat anti‐mouse IgG H&L (FITC) (ab6785) used for immunofluorescence analysis were from Abcam. Haematoxylin and eosin staining solution (BL700A‐1) was purchased from Biosharp. The tartrate‐resistant acid phosphatase (TRAP) staining kit (BB‐4421) was obtained from Bestbio. Bovine type II collagen (CII) solution (20022) were from Chondrex. GSK‐3β siRNA (sc‐270460), siRNA transfection medium (sc‐36868), siRNA transfection reagent (sc‐29528) and control siRNA (sc‐37007) were all purchased from Santa Cruz Biotechnology, Inc (Dallas, TX, USA).

### 2.2 Animals and groups

Thirty male C57BL/6J mice (6–8 weeks, weighing 18–20 g) were obtained from the Hubei Province Experimental Animal Center (Wuhan, China). All animals were housed in a 12 h light/dark circumstance with food and water ad libitum and allowed to acclimatize the environment for 5 days before the experiments. All the protocols have been approved by the Ethics Committee of Hubei University of Science and Technology (approval number: 2020‐01‐900). Animals were randomly divided into three groups (*n* = 10 for each group): control group, collagen‐induced arthritis (CIA) group and CIA + AB4 group.

### 2.3 Model establishment and drug administration

A CIA mouse model was established by intradermal injection of 20 μL CII mixture, which was homogenized and emulsified using 2 mg/mL CII solution and an equal volume of CFA on Day 0 and Day 7.^25^ AB4 was dissolved into DMSO at the stocking concentration of 100 mg/mL and was diluted in corn oil to the final concentration 10 mg/mL before used. A mixture of corn oil and DMSO (1:10) was used as vehicle. After CIA injection (Days 15, 16 and 17), CIA mice were intraperitoneally injected with vehicle and CIA + AB4 mice were injected with AB4 (10 mg/kg).

### 2.4 Behavioural tests

On Days 0, 7 and 14 after the CIA inducement, the behavioural tests were conducted on the mice, and 4 h after AB4 administration on Days 15–17. To detect the mechanical pain sensitivity, mouse was stimulated using test of the von Frey filaments (Stoelting, Wood Dale, USA) on the left hind paw after habituating. A brisk withdrawal and paw flinching was considered as the positive response. Contrary, no response was a negative response. The positive and negative withdrawal responses were converted into mechanical threshold through the formula.^26^ The numbers of flinches were recorded to present spontaneous pain.^27^ After habituating, the flinch numbers of mice were recorded within 5 min for three times. Latency to fall on rotarod was tested to present motor coordination.^28^ Mice were placed on rotarod at a constant speed at 10 rpm for 10 s and an increasing speed to 20 rpm for 30 s.

### 2.5 Tartrate‐resistant acid phosphatase (TRAP) staining

The animals were sacrificed and the left tibias were isolated and preserved in 10% neutral buffered formalin. After decalcifying in 10% EDTA for 21 days, embedding in paraffin and cutting, the 4‐μm tibia sections were stained with TRAP staining reagent for 1 h at 37°C and methyl green at 25°C temperature for 5–10 min. The images were captured by a microscope (IX73; Olympus).

### 2.6 Histology and immunofluorescence of the spinal cord

The mice were deeply anaesthetised (60 mg/kg sodium pentobarbital) and transcardially perfused with saline solution, followed by 4% paraformaldehyde. The lumbar spinal cord was collected and post‐fixed with 4% PFA overnight at 4°C. After embedding in paraffin, the spinal tissues were cut into 4‐μm sections and stained with the standard haematoxylin and eosin staining to observe histological changes and analysed using the ImageJ software. The inflammation cell infiltration scoring criteria are as follows^29^: 0 represents normal; 1 indicates lymphocyte infiltration around meninges and blood vessels; 2 represents 1–10 lymphocytes are observed in a field; 3 and 4 represents 11–100 lymphocytes and more than 100 lymphocytes are observed in a field, respectively.

Immunofluorescence was used to detect the expression and localization of proteins. After dewaxing, conducting to antigen retrieval, treating with 3% hydrogen peroxide, and blocking with immunofluorescence blocking solution, the sections of the spinal cord were incubated with primary antibodies (1:100) overnight at 4°C and fluorescent secondary antibody at the dilution of 1:1000 for 1 h, observed under a fluorescence microscope and analysed using the ImageJ software.

### 2.7 Transmission electron microscopy (TEM)

Mitochondrion in the spinal cord was confirmed by electron microscopy. Briefly, about 1 mm^3^ cubes of spinal cord tissues were fixed in 2.5% glutaraldehyde, cut into 4‐μm sections following with staining by uranyl acetate and lead citrate, and examining by an HC‐1 transmission electron microscope (Hitachi, Tokyo, Japan).

### 2.8 Western blotting assays

Mice were sacrificed after behavioural tests, the lumbar spinal cords were obtained and homogenized on ice in RIPA lysis buffer (containing 1% protease inhibitors), centrifuged at 12,000 g, 4°C for 20 min and collected the supernatant. After determination of protein concentration, the samples were separated on SDS–PAGE gel and transferred to PVDF membranes, which were blocked with blocking buffer for 60–90 min, incubated with the primary antibodies (1:1000) overnight at 4°C and HRP‐conjugated secondary antibodies (1:10,000) for 1 h, probed with an ECL detection reagent, visualized using an iBright 1500 instrument (Invitrogen) and analysed by the ImageJ software.

### 2.9 Molecular docking

The structure of glycogen synthase kinase‐3β (GSK‐3β) was obtained from the Protein Data Bank (PDB ID: 1H8F). The structure of AB4 was downloaded from the PubChem database (PubChem CID: 71307558). The conformation between GSK‐3β and AB4 was docked using the Auto Dock Vina 1.2.0 software and visualized by the PyMOL 2.2.3 software.

### 2.10 Isothermal titration calorimetry (ITC) assay

ITC experiment on the interaction of AB4 with GSK‐3β (50650‐M07B, SinoBiological, China) were carried out at 25°C using an iTC_200_ titration calorimetry (MicroCal, Northampton, MA). GSK‐3β was diluted in 20 mM Tris‐HCl buffer (pH 7.4) at 10 μM, and loaded into the sample cell (200 μL). A solution of 100 μM AB4 was placed in the injection syringe (40 μL). The ITC titration data are fitted to a single set of identical sites model using the MicroCal ORIGIN software supplied with the instrument.

### 2.11 Superoxide dismutase (SOD) activity measurement

A SOD assay kit was used to analyse the SOD enzyme activity. Briefly, after homogenizing in PBS and centrifuging at 12,000 g for 15 min, the supernatant was collected and incubated with the WST‐8 enzyme working solution for 20 min at 37°C, and measured the OD_450 nm_ absorbance.

### 2.12 Cell preparation and treatment

The penicillin‐streptomycin mixture (Gibco, 15070063) were added in the medium, the final concentration of penicillin is 50 U/mL and the streptomycin is 50 μg/mL. C6 cells were induced with 5 ng/μL IL‐1β for 4 h, combing with 0 or 1 μM AB4 treatment for 24 h.

### 2.13 Mitochondrial membrane potential (MMP) measurement

JC‐1 MMP assay kit and Mito‐Tracker Red CMXRos were used to evaluate the MMP. According to the instructions, after IL‐1β inducement and AB4 treatment, cells were washed thrice with PBS, cultured with JC‐1 or Mito‐Tracker Red CMXRos for 20 min at 37°C in the dark and observed under fluorescence microscope.

### 2.14 Mitochondrial lipid peroxidation assessment

3‐[4‐(Perylenylphenylphosphino) phenoxy] propyltriphenylphosphonium iodide (MitoPeDPP) (M466, Dojindo Molecular Technologies, Inc.) is a cell‐membrane‐permeable probe that can penetrate the cell membrane and aggregate in mitochondria, and used to assess lipid peroxidation in mitochondria. After IL‐1β inducement and AB4 treatment, the cells were washed thrice with PBS, stained with 0.3 μmol/L MitoPeDPP and 10 μmol/L DAPI in the dark at 37°C for 15 min and detected under fluorescence microscope.

### 2.15 Cytosolic ROS assessment

Dihydroethidium (DHE) probe was used to investigate the cytosolic ROS levels in cells. DHE produce red fluorescence by interacting with ROS. The cells were induced by IL‐1β, treated with AB4, washed thrice with PBS, stained with 10 μmol/L DHE solution for 30 min in dark and detected under a fluorescence microscope.

### 2.16 Transient transfection

Chemically synthesized GSK‐3β siRNA targeting GSK‐3β was purchased from Santa Cruz Biotechnology, Inc. C6 cells were seeded in six‐well culture dishes with 2 mL antibiotic‐free DMEM/F12 medium that was supplemented with FBS. At 30%–50% confluency, the cells were transfected with GSK‐3β siRNA and control siRNA for 7 h at 37°C. After incubation, 1 mL DMEM/F12 medium was added to each well without removing the transfection mixture; the cells were incubated for an additional 24 h. Negative control siRNA was used to assess non‐specific gene‐silencing effects. GSK‐3β level in C6 cells were assessed by western blotting after transient transfection.

### 2.17 Statistical analysis

All statistical analyses were performed by the SPSS 26.0 software. Data for paw thickness and ankle widen were analysed by student's *t*‐test. The other data were analysed by one‐way analysis of variance followed by Tukey's test. Data for haematoxylin and eosin staining, immunofluorescence and western blotting were presented as mean ± SD. Data for behaviour tests were expressed as mean ± SEM. *p* < 0.05 showed the statistical significance.

## 3 RESULTS

### 3.1 AB4 relieves CIA‐induced pain hypersensitivity

**FIGURE 1:**
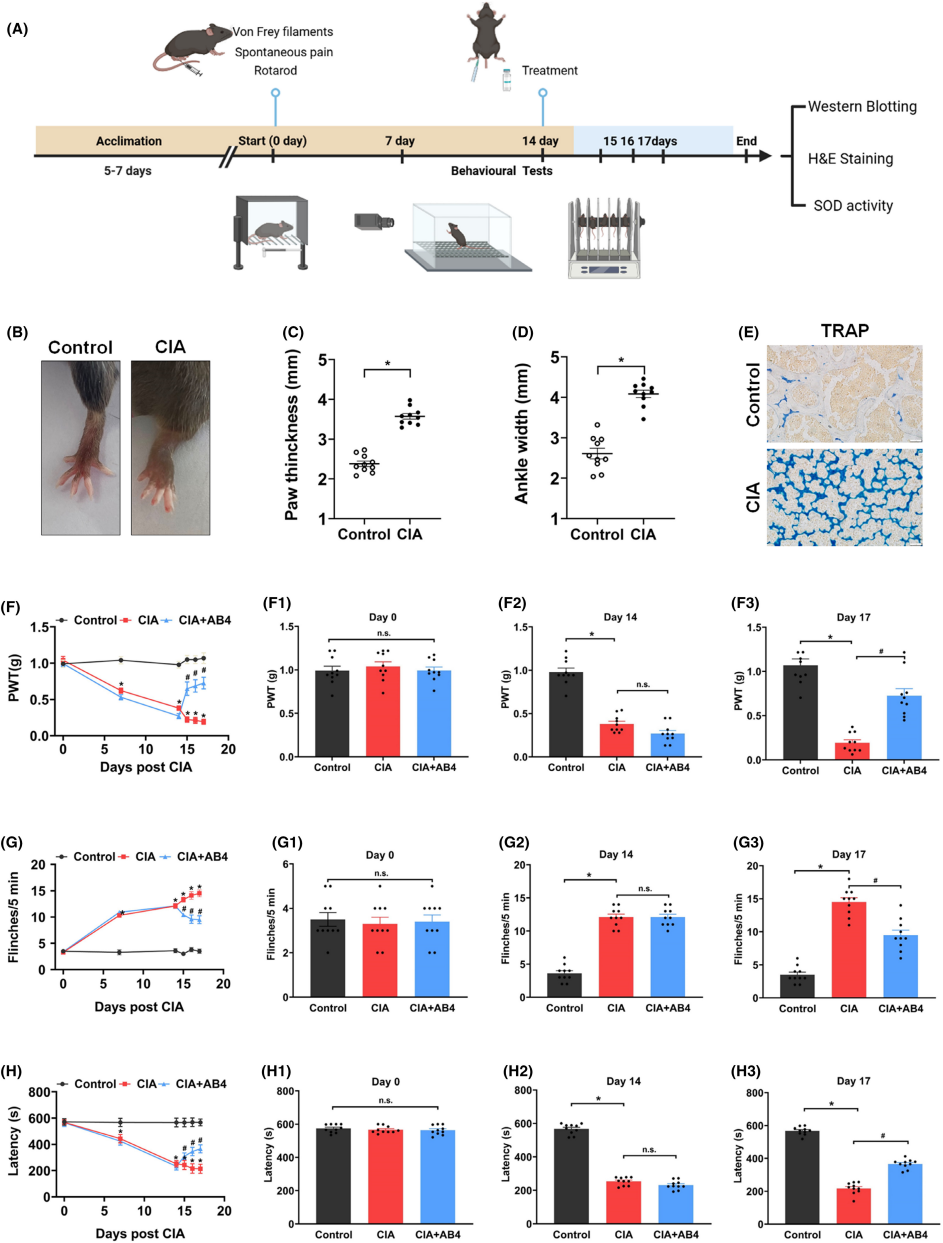
Effect of AB4 treatment on pain response in CIA‐induced mice. (A) Schematic diagram of the experimental procedures. CIA was intraarticularly injected into the left knee joint of mice on Day 0. Behavioural tests were performed on Days 0, 7 and 14. AB4 was intraperitoneally injected in mice on Days 15–17. Behavioural tests were performed after 4 h of AB4 treatment. Then, mice were scarified and the spinal cord tissues were collected for morphological and expression analyses. (B) Representative images of the left hind paw from control and CIA mice. (C, D) Changes of paw thickness (C) and ankle width (D) from control and CIA mice. (E) Representative tibia areas stained with TRAP and methyl green. Scale bar = 20 μm. (F–H) changes of PWT values (F), spontaneous flinches (G) and latency to fall (H) of mice. Histogram analysis for PWT values (F1–F3), spontaneous flinches (G1–G3) and latency to fall (H1–H3) at 0, 14 and 17 days. Data are expressed as the mean ± SEM (*n* = 10). *p < 0.05 versus control group, ^#^ *p* < 0.05 versus CIA group.

Behaviour tests were performed as the protocols shown in Figure 1A. Figure 1B shows the swelled paw and increased ankle width were observed after 14‐day CIA induction (*p* < 0.05 vs. control group, Figure 1C,D). Meanwhile, histology revealed that the TRAP‐positive cells presenting osteoclast‐like cells with were clearly visible within the bone matrix in the CIA group (Figure 1E). Next, the mechanical pain was detected, the pain thresholds were significantly decreased in the CIA group. Moreover, the numbers of flinches were dramatically increased in the CIA group. Latency to fall were also reduced in the CIA group. On Days 15, 16 and 17, AB4 treatment statistically raised mechanical threshold values, descended flinches numbers and increased latency to fall (*p* < 0.05 vs. CIA group). These results indicated that AB4 treatment decreased pain hypersensitivity and enhanced motor coordination in CIA mice.

### 3.2 AB4 reduces NLRP3‐mediated inflammation in the spinal cord of CIA mice

**FIGURE 2:**
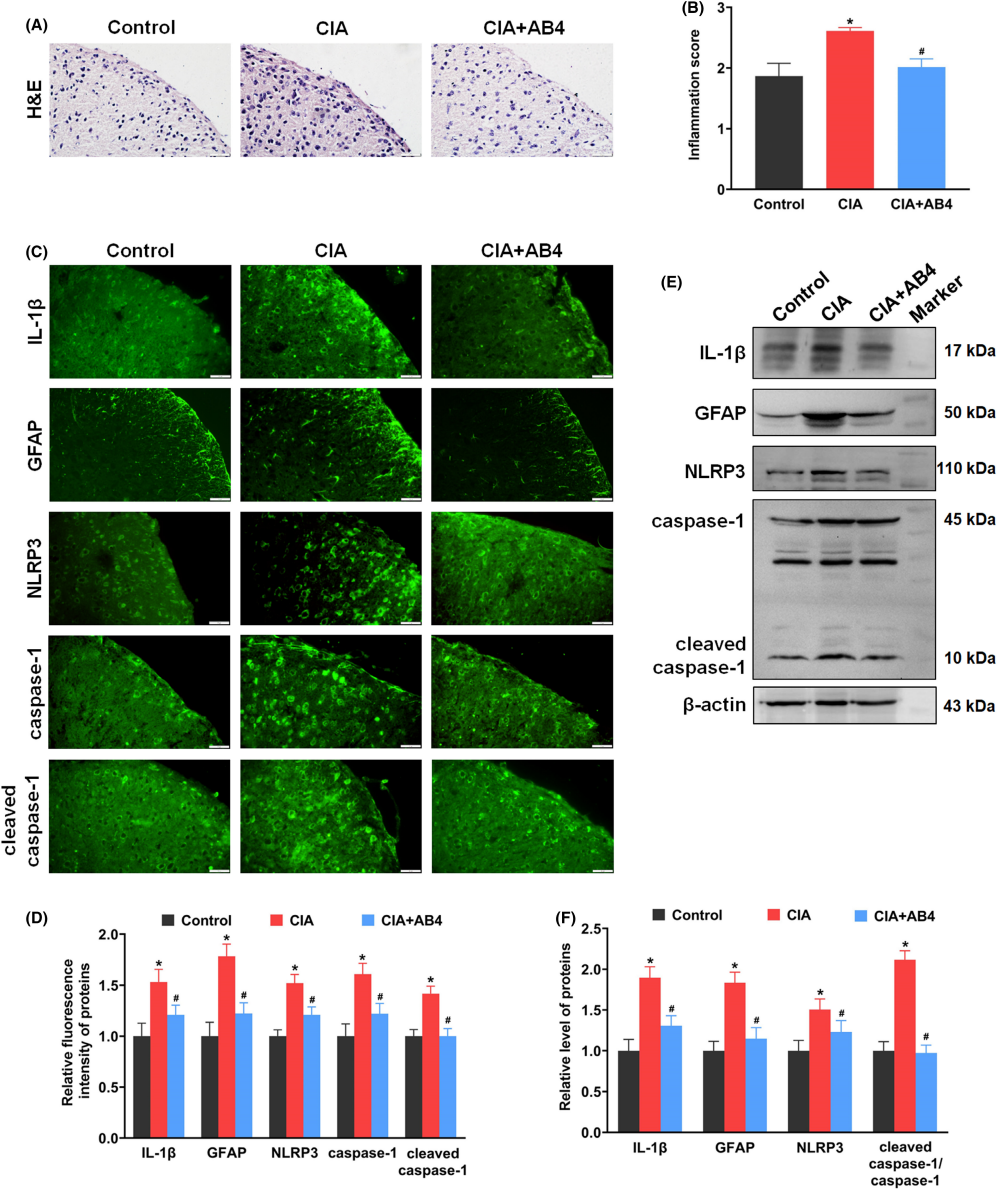
Effects of AB4 treatment on inflammatory infiltration and protein levels of IL‐1β, GFAP, NLRP3 and caspase‐1 in the spinal cord of the control, CIA and CIA + AB4 groups. (A, B) Representative haematoxylin and eosin staining images (A) and quantitative analysis (B) of the spinal cord sections. Scale bar = 20 μm. (C) Representative immunofluorescence staining images of IL‐1β, GFAP, NLRP3, caspase‐1 and cleaved caspase‐1 in the spinal dorsal horn. Scale bar = 20 μm. (D) Quantitative analysis of the fluorescence intensity of IL‐1β, GFAP, NLRP3, caspase‐1 and cleaved caspase‐1. (E, F) Western blot analysis and quantitative grey value analysis of IL‐1β, GFAP, NLRP3, caspase‐1 and cleaved caspase‐1 levels in the spinal cord of the control, CIA and CIA + AB4 groups. Data are presented as mean ± SD (*n* = 5). *p < 0.05 versus control group, ^#^ *p* < 0.05 versus CIA group.

Histological analysis showed severe inflammatory cells infiltration in the spinal dorsal horn of CIA mice (*p* < 0.05 vs. control group, Figure 2A,B). Protein localization and expression analysis showed enhanced fluorescence intensity and upregulated protein expression of IL‐1β in the spinal dorsal horn of the CIA group (*p* < 0.05 vs. control group, Figure 2C–F). Activated astrocyte is a main source of proinflammation cytokines, marked with protein GFAP.^11^ Consistent with the increased inflammatory response, the intensity and protein expression of GFAP were both dramatically increased in the CIA group (*p* < 0.05 vs. control group, Figure 2C–F). Next, the NLRP3 inflammasome‐mediated signalling were detected. There was increase in the fluorescence intensity of NLRP3 inflammasome components (NLRP3 and caspase‐1) in the CIA group. The protein expression analysis also proved the upregulated level of NLRP3 in the CIA group (*p* < 0.05 vs. control group, Figure 2C–F). After AB4 treatment, the inflammatory response in the spinal cord was decreased in the CIA + AB4 group (*p* < 0.05 vs. CIA group, Figure 2A,B). The intensity and protein expression of spinal IL‐1β, GFAP, NLRP3, caspase‐1 and cleaved caspase‐1 were reduced in the CIA + AB4 group (*p* < 0.05 vs. CIA group, Figure 2C–F).

### 3.3 AB4 stimulated Nrf2‐mediated antioxidative response in the spinal cord

**FIGURE 3:**
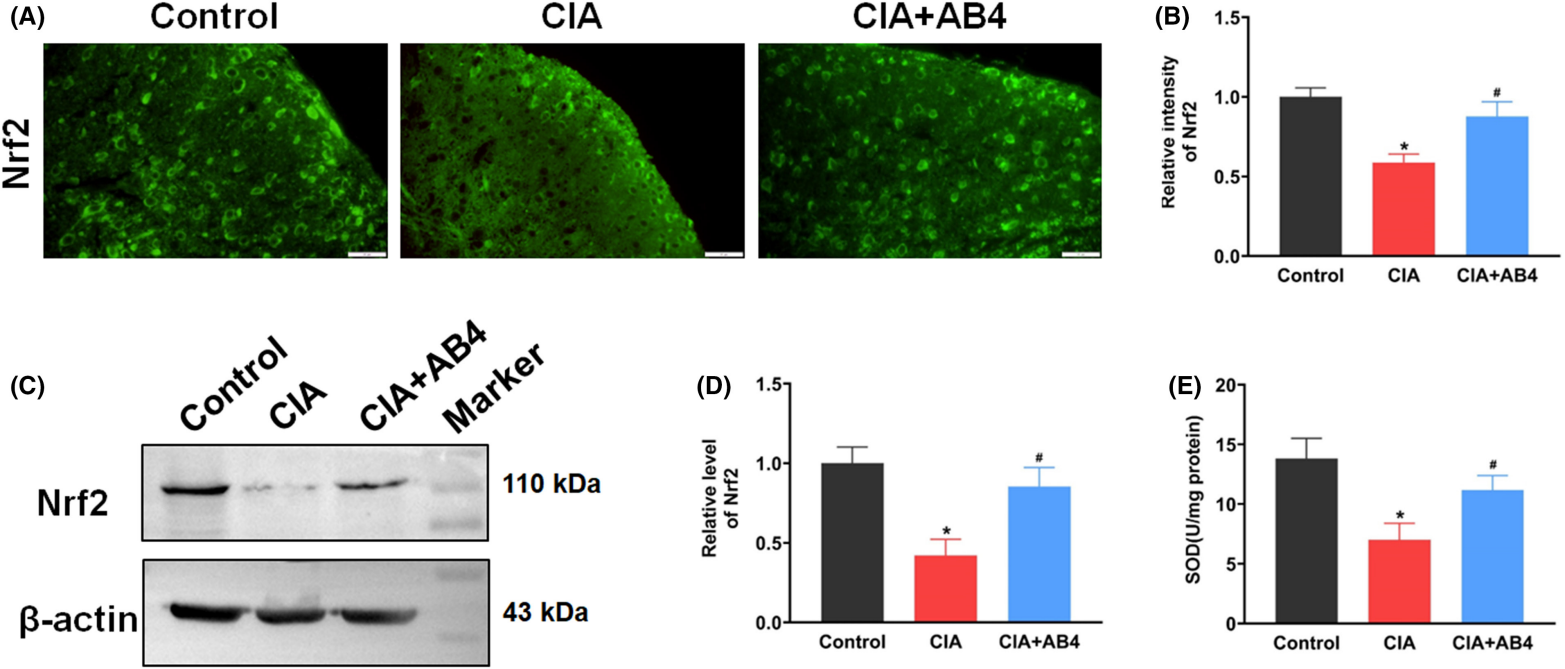
Effect of AB4 treatment on Nrf2 expression and SOD activity in the spinal cord of mice. (A, B) Representative immunofluorescence staining images (A) and quantitative intensity analysis (B) of Nrf2 in the spinal dorsal horn of the control, CIA and CIA + AB4 groups. Scale bar = 20 μm. (C, D) Western blot analysis and quantitative grey value analysis of Nrf2 level in the spinal cord of the control, CIA and CIA + AB4 groups. (E) SOD activity in the spinal cord of the control, CIA and CIA + AB4 groups. Data are presented as mean ± SD (*n* = 5). *p < 0.05 versus control group, ^#^ *p* < 0.05 versus CIA group.

Oxidative stress directly and indirectly promotes NLRP3 inflammasome activation,^30^ Nrf2 expression and SOD activity as the antioxidative elements were detected. The intensity and expression of spinal Nrf2 was decreased in the CIA group (*p* < 0.05 vs. control group) and AB4 treatment increased Nrf2 intensity (*p* < 0.05 vs. CIA group, Figure 3A,B) and upregulated the Nrf2 level (*p* < 0.05 vs. CIA group, Figure 3C,D). Meanwhile, in contrast to the control group (13.83 ± 1.69 U/mg), SOD activity was significantly suppressed in the CIA group (7.01 ± 1.40 U/mg, *p* < 0.05 vs. control group), while AB4 treatment increased the SOD activity (11.18 ± 1.22 U/mg, *p* < 0.05 vs. CIA group, Figure 3E).

### 3.4 AB4 restores mitochondrial function in the spinal cord of CIA mice

Mitochondrial dysfunction plays key role in chronic pain by connecting with oxidative stress and inflammation.^31^

**FIGURE 4:**
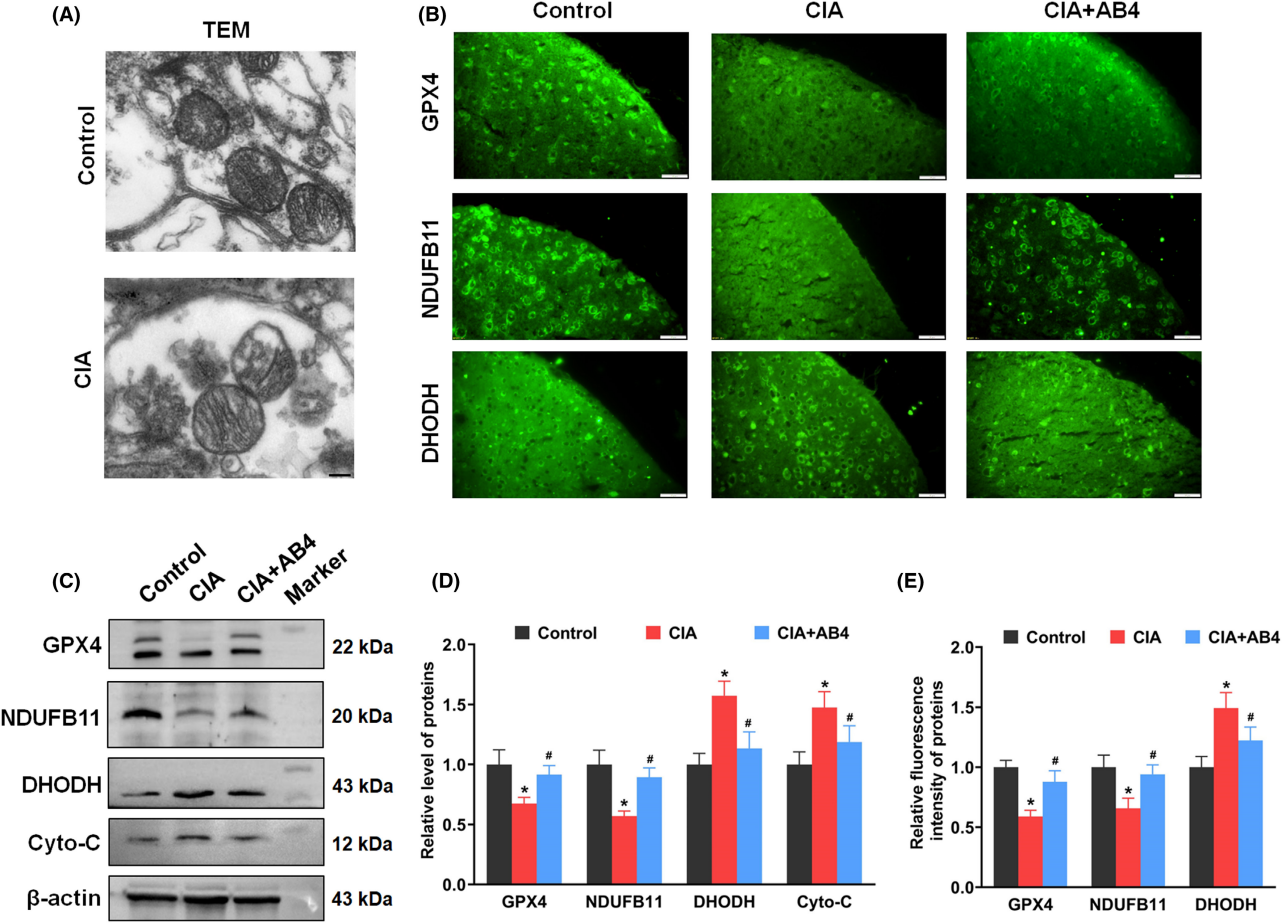
Effect of AB4 treatment on mitochondrial function in the spinal cord of mice. (A) Representative TEM images of spinal mitochondria from control and CIA mice. Scale bar = 200 nm. (B) Representative immunofluorescence staining images of GPX4, NDUFB11 and DHODH, in the spinal dorsal horn of the control, CIA and CIA + AB4 groups. Scale bar = 20 μm. (C) Quantitative analysis of the fluorescence intensity of GPX4, NDUFB11 and DHODH. (C) (D, E) Western blot analysis and quantification of the relative grey value of GPX4, NDUFB11, DHODH and Cyto‐C expression level in the spinal cord of the control, CIA and CIA + AB4 groups. Data are presented as mean ± SD (*n* = 5). *p < 0.05 versus control group, ^#^ *p* < 0.05 versus CIA group.

Morphology of mitochondrial was detected by TEM. Normal outer‐membrane and cristae of mitochondrial structure was observed in the control group. Conversely, mitochondria structure exhibited an increased outer membrane density, and a disappearance and disrupted cristae in the CIA group (Figure 4A). GPX4 is modulated by Nrf2 and has a major role in protecting mitochondria from oxidative damage.^32^, ^33^ As a small mitochondrial integral membrane protein NDUFB11 is essential for the active complex I assembly in mitochondrial respiratory chain.^34^ As an antioxidant, DHODH inhibits mitochondrial lipid peroxidation and protects cells from ferroptosis.^35^ As shown in Figure 4B,E, the fluorescence intensity of GPX4 and NDUFB11 was significantly reduced, while the intensity of DHODH was significantly enhanced in the spinal cord of CIA mice (*p* < 0.05 vs. control group). AB4 treatment increased the fluorescence intensity of GPX4 and NDUFB11 and reduce the intensity of DHODH in the spinal cord of CIA + AB4 mice (*p* < 0.05 vs. CIA group, Figure 4B,E). For protein expression analysis, CIA inducement significantly reduced spinal GPX4 and NDUFB11 protein levels while CIA inducement increased DHODH and cyto‐c protein levels (*p* < 0.05 vs. control group, Figure 4C,D). AB4 administration restored the disordered changes in expression of GPX4, NDUFB11, DHODH and cyto‐c in the spinal cord of CIA + AB4 mice (*p* < 0.05 vs. CIA group, Figure 4C,D).

### 3.5 AB4 decreases spinal GSK‐3β/Drp1 signalling activity

**FIGURE 5:**
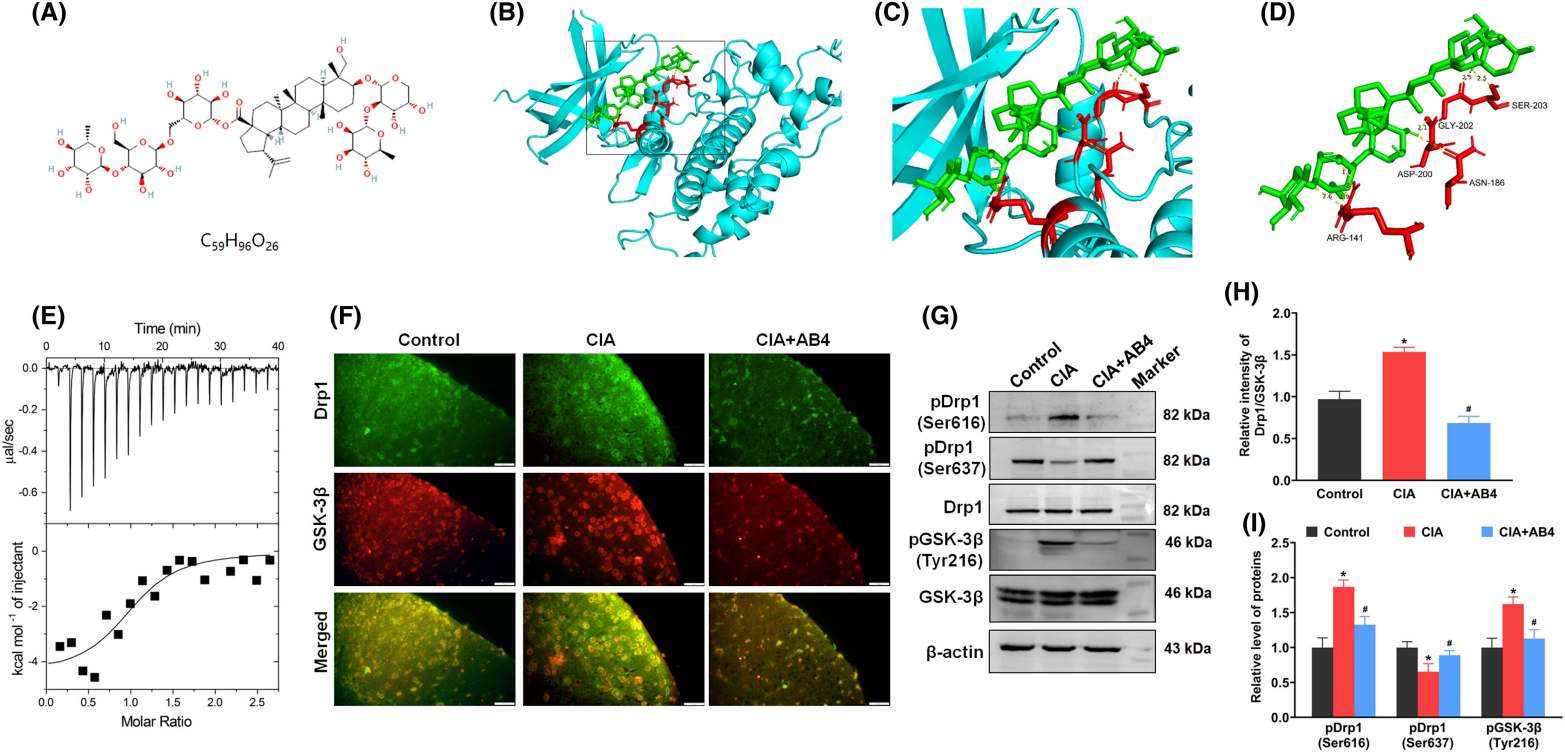
(A) The molecular structure of AB4. (B–D) Molecular docking of AB4 with GSK‐3β. The modelled 3D structure of GSK‐3β docked with AB4 (B). The enlarged view of binding site in box (C). The interaction bonds of GSK‐3β with AB4 (D). Bonds showed as yellow dotted lines, and bond lengths were presented as numbers. (E) The titration between AB4 and GSK‐3β. The top panel presents typical calorimetric titration of AB4 with GSK‐3β at 25°C. The bottom panel shows the plots of the heat evolved (kcal) per mol of AB4 added corrected for the heat of with GSK‐3β, against the molar ratio of AB4 to GSK‐3β. Data solid squares were fitted to a single set of the identical sites model, and the solid line represented the best fit. (F) Representative immunofluorescence staining images of GSK‐3β and Drp1 in the spinal dorsal horn of the control, CIA and CIA + AB4 groups. Scale bar = 20 μm. (G) Quantitative analysis of the fluorescence intensity of GSK‐3β and Drp1. (H, I) Western blot analysis and quantitative grey value analysis of pGSK‐3β‐Tyr216, GSK‐3β, pDrp1‐Ser616, pDrp1‐Ser637 and Drp1 level in the spinal cord of the control, CIA and CIA + AB4 groups. Data are presented as mean ± SD (*n* = 5). *p < 0.05 versus control group, ^#^ *p* < 0.05 versus CIA group.

GSK‐3β is considered as a potential target for pain management and GSK‐3β inhibition potently promotes ferroptotic resistance.^36^ In order to detect the binding affinity of AB4 on GSK‐3β, the molecular docking assay estimated the GSK‐3β x‐ray crystal structures and the ligand AB4. The molecular structure and formula of AB4 (PubChem CID: 71307558) are shown in Figure 5A. The molecular docking analysis showed that AB4 formed five electrovalent bonds with GSK‐3β at Arg‐141, Asp‐200, Asn‐186, Gly‐202 and Ser‐203 with the binding affinity at −9.1 kcal/mol (Figure 5B–D). To characterize the interaction between AB4 and GSK‐3β, ITC experiments are carried out. The raw titration curve presenting as the negative peaks in the plots of power versus time was observed for heat generation, which suggested that there was an interaction between AB4 and GSK‐3β. The fitted binding affinity was 5.6 ± 19 μmol/L (Figure 5E). Meanwhile, the intensity of GSK‐3β in the spinal cord of CIA group was increased (*p* < 0.05 vs. control group, Figure 5F,H), while AB4 administration decreased the intensity of GSK‐3β (*p* < 0.05 vs. CIA group, Figure 5F,H). The expression level of pGSK‐3β Tyr216 as an active site for GSK‐3β^37^ which was upregulated in the CIA group (*P* < 0.05 vs. control group, Figure 5G,I) and was reduced following AB4 treatment (*p* < 0.05 vs. CIA group, Figure 5G,I).

It is reported that GSK‐3β activation increases Drp1 GTPase activity and promotes mitochondrial fission via regulating Drp1 phosphorylation.^38^ Compared to control group, CIA inducement increased the intensity of spinal Drp1 (*p* < 0.05 vs. control group), upregulated the phosphorylation of Drp1 at Ser616 (*p* < 0.05 vs. control group) and downregulated phosphorylation of Drp1 at Ser637 (*p* < 0.05 vs. control group). AB4 treatment recovered the intensity and protein level of Drp1 (Figure 5F–I).

### 3.6 AB4 recovers mitochondrial function and mitochondrial ROS levels in C6 cells

**FIGURE 6:**
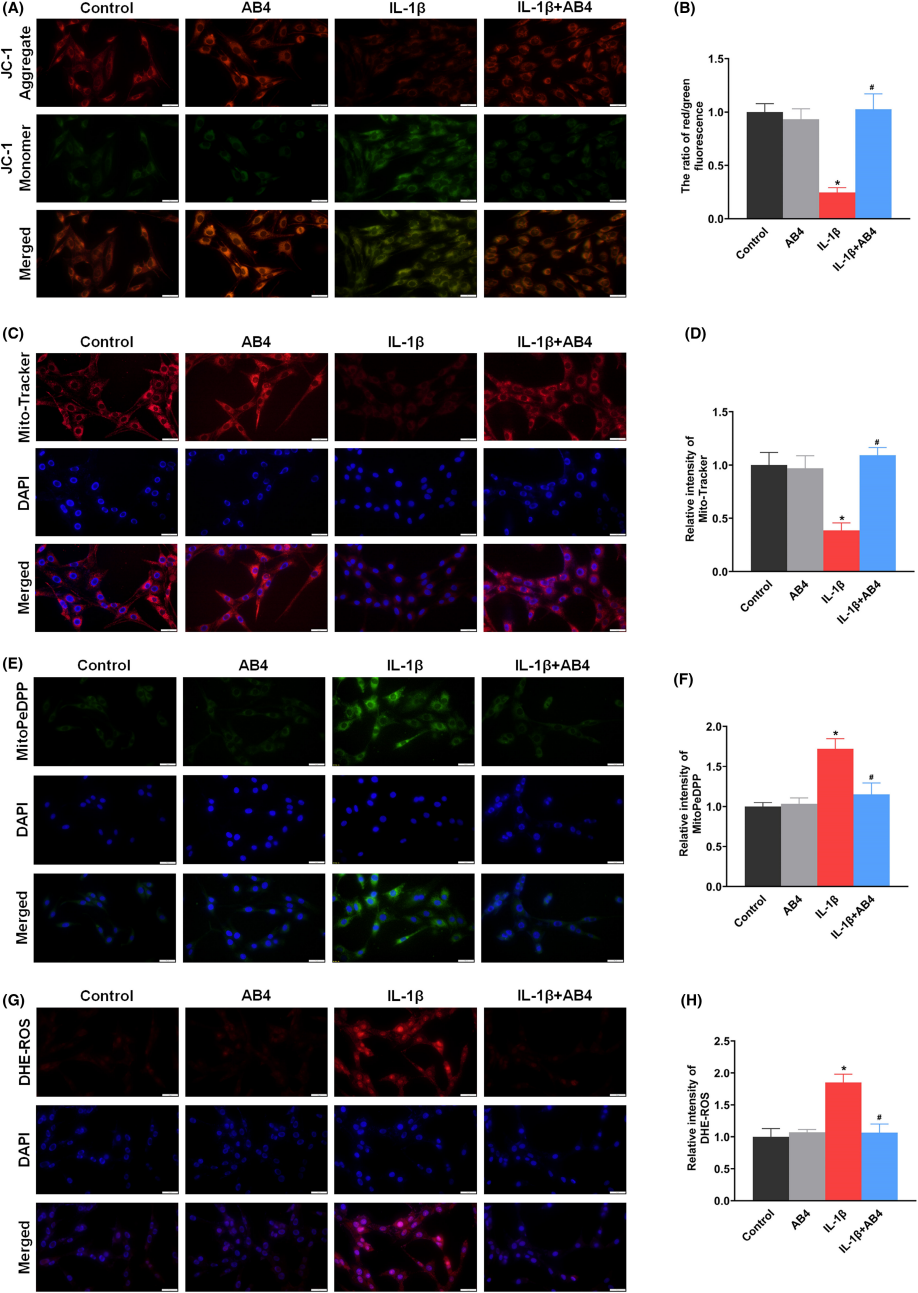
Effect of AB4 treatment on the mitochondrial membrane potential, mitochondrial lipid peroxide and cellular ROS in C6 cells. (A, B) Representative fluorescence images (A) and quantitative analysis (B) of JC‐1 in the control, AB4, IL‐1β and IL‐1β + AB4 groups. (C, D) Representative fluorescence images (C) and quantitative analysis (D) of Mito‐Tracker in the control, AB4, IL‐1β and IL‐1β + AB4 groups. (E, F) Representative fluorescence images (E) and quantitative analysis (F) of MitoPeDPP in the control, AB4, IL‐1β and IL‐1β + AB4 groups. (G, H) Representative fluorescence images (I) and quantitative analysis (J) of DHE in the control, AB4, IL‐1β and IL‐1β + AB4 groups. Scale bar = 20 μm. Data are expressed as the mean ± SD (*n* = 5). *p < 0.05 versus control group, ^#^ *p* < 0.05 versus IL‐1β group.

To investigate the effects of AB4 on mitochondrial function and ROS level, changes in MMP were determined using JC‐1 indicator and Mito‐Tracker Red assay. JC‐1 aggregates and Mito‐Tracker represents higher MMP, and emits as red fluorescence, whereas JC‐1 monomer represents lower MMP and emits as green fluorescence.^39^ As shown in Figure 6A,B, IL‐1β stimulation decreased the relative ratio of red to green fluorescence (*p* < 0.05 vs. control group group). AB4 treatment increased the ratio of red to green fluorescence (*p* < 0.05 vs. IL‐1β group). Meanwhile, the relative intensity of Mito‐Tracker was reduced after IL‐1β stimulation, respectively (*p* < 0.05 vs. control group) while AB4 treatment recovered fluorescence intensity (*p* < 0.05 vs. IL‐1β group, Figure 6C,D). The results indicated that AB4 attenuates mitochondrial dysfunction induced by IL‐1β induction.

Mitochondrial lipid peroxide changes were detected using MitoPeDPP which presents as the green fluorescence and binds with lipid peroxide in the mitochondrial inner membrane.^40^ The level of mitochondrial lipid peroxide was significantly increased after IL‐1β stimulation, represented by increased fluorescence intensity (*p* < 0.05 vs. control group). AB4 treatment decreased the mitochondrial lipid peroxide level (*p* < 0.05 vs. IL‐1β group, Figure 6E,F). Furthermore, cellular ROS level was detected using DHE dying. The cellular ROS was significantly increased after IL‐1β or erastin stimulation (*p* < 0.05 vs. control group) and this increase was abated by AB4 treatment (*p* < 0.05 vs. IL‐1β group, Figure 6G,H). However, AB4 had little effect on the control cells.

### 3.7 AB4 decreased the expression of GSK‐3β and Drp1 levels in IL‐1β C6 cells

**FIGURE 7:**
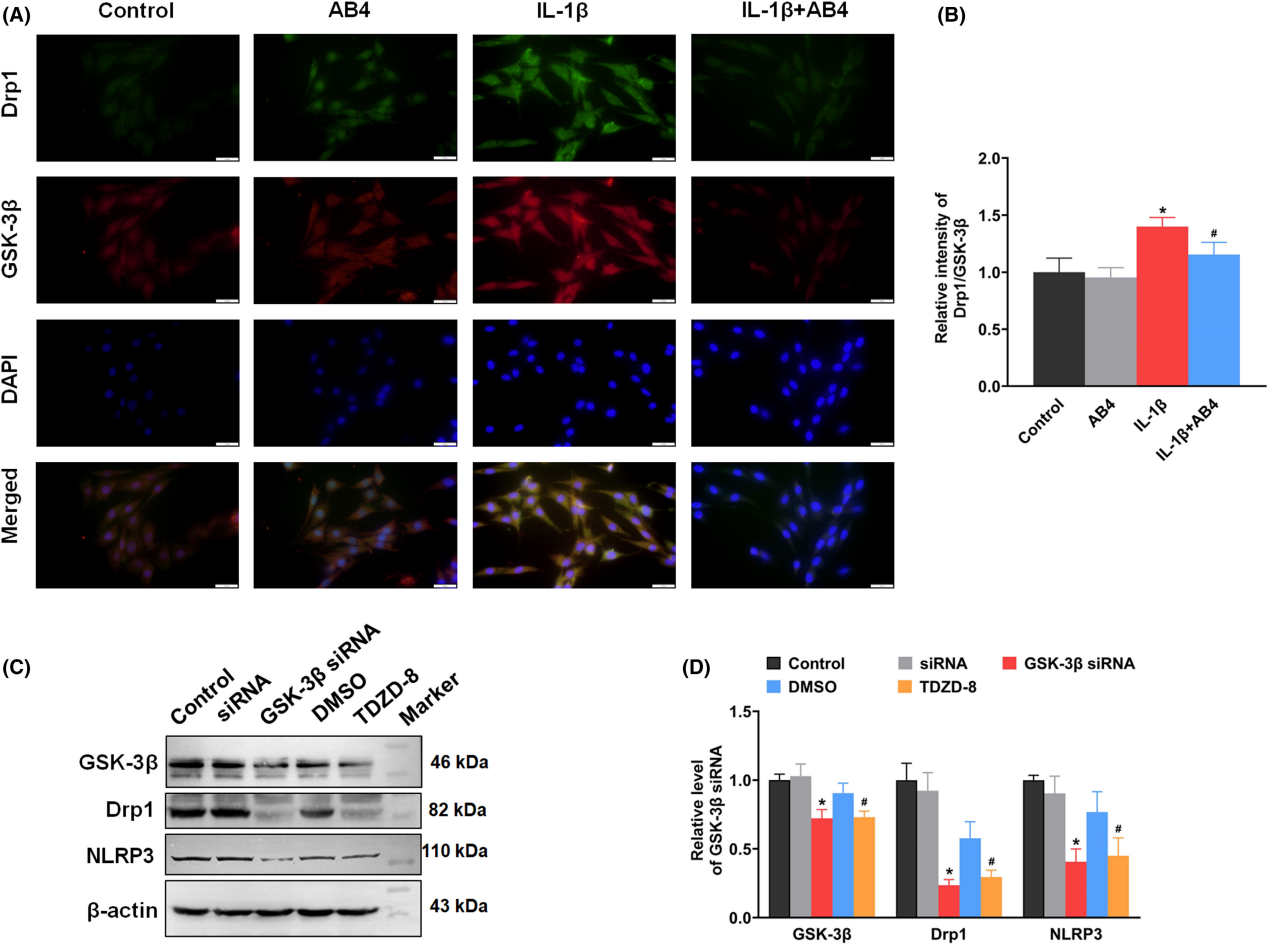
Effect of AB4 treatment on GSK‐3β and Drp1 expression in C6 cell. Representative immunofluorescence staining images (A) and quantitative analysis (B) for Drp1 and GSK‐3β in C6 cells. Cells were counterstained with Hoechst 33342 (blue). Scale bar = 20 μm. Data were presented as mean ± SD (*n* = 5). *p < 0.05 versus control group. ^#^ *p* < 0.05 versus IL‐1β group. (C–D) Western blotting measured the protein levels of GSK‐3β, Drp1 and NLRP3 in C6 cells transfected with GSK‐3β siRNA or treated with TDZD‐8. Relative protein levels are expressed as fold changes over control. Data were presented as mean ± SD (*n* = 3). *p < 0.05 versus siRNA group, ^#^ *p* < 0.05 versus DMSO group.

The colocalization of GSK‐3β and Drp1 in C6 cells was measured by double immunofluorescence staining. Compared with the control group, signal intensities of GSK‐3β and Drp1 were increased upon IL‐1β induction and AB4 treatment reduced the intensities (Figure. 7A,B). To further confirm the correlation of GSK‐3β and Drp1, GSK‐3β siRNA was used to knockdown its expression level. As shown in Figure 7C, GSK‐3β siRNA induced the decrease in Drp1 protein level. We also detected the NLRP3 level and found that GSK‐3β knockdown reduced the NLRP3 protein level. Moreover, we also used GSK‐3β inhibitor TDZD‐8 to treat C6 cell and the decreased protein levels of GSK‐3β, Drp1 and NLRP3 were observed. The results indicated that changes in GSK‐3β expression and activity would lead to the abnormal expression of Drp1.

## 4 DISCUSSION

In our study, we found that AB4 treatment alleviated arthritis pain by inhibiting GSK‐3β activity. GSK‐3β plays a crucial role in pain. In rodent animal models of neuropathic pain, GSK‐3β activity is increased.^41^ Suppressing GSK‐3β activity by ghrelin and TDZD‐8 in the spinal dorsal horn attenuates the release of pro‐inflammatory cytokines and alleviates pain sensitivity.^42^ Meanwhile, GSK‐3β inhibition also suppresses β‐catenin pathway activity, attenuates the apoptosis, and facilitates knee function in osteoarthritis model.^43^ Here, we found AB4 administration docked with GSK‐3β via kinase domain. Moreover, AB4 administration inhibited GSK‐3β activity by reducing pGSK‐3β Tyr216 level. Accordingly, we suggested that AB4 alleviated pain via inhibiting GSK‐3β activity.

AB4 was found to suppress neuroinflammation in the spinal cord and alleviate pain via GSK‐3β/Nrf2 signalling pathways in our study. Nrf2 acts as a protective regulator for inflammation and chronic pain. Nrf2 activation directly blocks the transcription of the proinflammatory cytokines and inflammatory mediators, including tumour necrosis factor (TNF), IL‐1β, IL‐6 and COX‐2.^44^, ^45^ As a protective regulator, Nrf2 acts against activation of NLRP3 inflammasome by regulating the thioredoxin1/thioredoxin interacting protein complex,^46^ and scavenging ROS.^47^ Moreover, Nrf2 regulates ferroptosis via controlling the components of the glutathione and thioredoxin antioxidant systems at the transcriptional level,^48^ and directly or indirectly regulating GPX4 expression and function.^49^ Increasing Nrf2/GPX4 signalling enhances SOD levels, inhibits ferroptosis and proinflammatory cytokines levels.^50^ Conversely, Nrf2 deficiency causes inflammatory exacerbation.^44^ Additionally, Nrf2 activation attenuates chronic pain in the model of chronic constriction injury.^29^, ^51^ GSK‐3β phosphorylates Nrf2 at Neh6 domain, initiates the proteasomal degradation of Nrf2.^52^ GSK‐3β inhibition reduces nuclear export, degradation of Nrf2^53^ and upregulates levels of Nrf2 expression and its downstream genes.^54^ In our study, AB4 elevated Nrf2/GPX4 signal, reduced ROS levels and NLRP3 inflammasome activation. Taken together, it was considered that AB4 treatment enhanced antioxidant response and suppressed inflammation via inhibiting GSK‐3β activity.

AB4 stimulated antioxidant response via GSK‐3β/Drp1 signalling. Complexes of electron transport chain in mitochondria have been identified as the most producing area of ROS.^55^ Mitochondrion are dynamic organelles whose distribution quantity and morphology changes depending on energy requirements.^56^ During chronic pain processing, mitochondrial fragment and dysfunction are induced in the spinal cord, which consequently lead to mitochondrial ROS release.^17^ GSK‐3β plays a key role in maintaining mitochondrial function. GSK‐3β causes abnormal mitochondrial fission by inducing Drp1‐S616 phosphorylation.^57^ GSK‐3β regulates the complex I, II, III and IV activity in mitochondrial respiratory chain and involves in ATP and ROS production.^58^ GSK‐3β phosphorylates voltage‐dependent anion channel to affect MMP.^59^ In our study, AB4 treatment reduced mitochondrial ROS levels in the spinal cord of rats and in C6 cells. These results suggested that AB4 treatment reduced mitochondrial and cellular ROS levels via inhibiting GSK‐3β activity.

## 5 CONCLUSION

**FIGURE 8:**
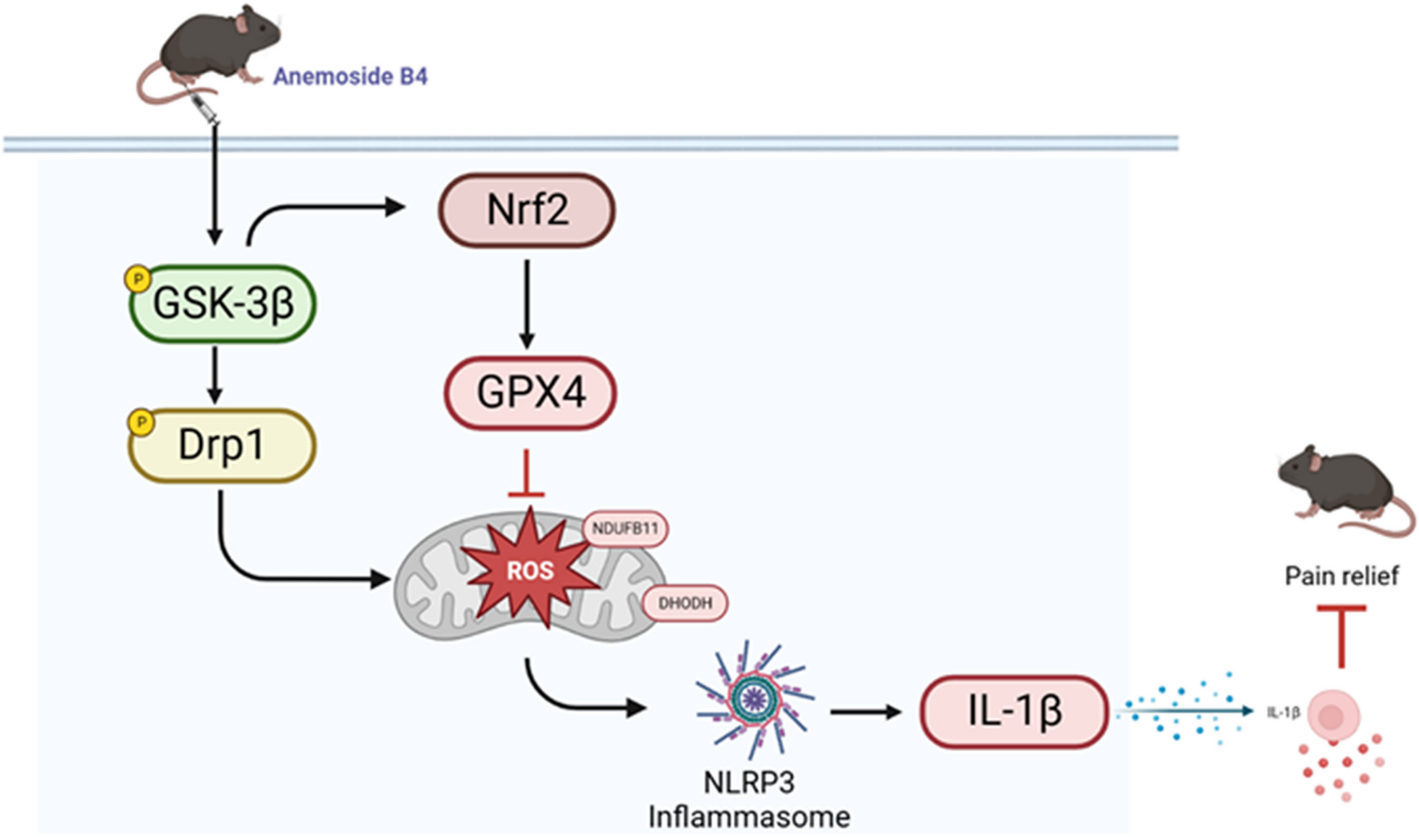
Schematic representation of the potential mechanisms of AB4 on arthritis pain management. AB4 inhibits GSK‐3β, increases Nrf2/GPX4‐mediated antioxidant response, reduces Drp1‐mediated mitochondrial dysfunction, suppresses inflammation and relives arthritis pain.

During RA processing, spinal inflammation is stimulated, ferroptosis is enhanced, and chronic pain is induced. AB4 treatment inhibits spinal GSK‐3β activity, enhances Nrf2/GPX4 antioxidant response, reduces Drp1‐mediated mitochondrial ROS production, suppresses NLRP3 inflammasome activation and alleviates arthritis pain (Figure 8).

## AUTHOR CONTRIBUTIONS

**Chenlu Guo:** Data curation (lead); formal analysis (equal); methodology (equal); validation (equal); visualization (equal). **Yuanfen Yue:** Data curation (equal); formal analysis (equal); methodology (equal). **Bojun Wang:** Data curation (equal); methodology (equal). **Shaohui Chen:** Data curation (equal); investigation (equal). **Dai Li:** Supervision (equal). **Fangshou Zhen:** Supervision (equal). **Ling Liu:** Methodology (equal). **Haili Zhu:** Conceptualization (equal); writing – original draft (equal). **Min Xie:** Conceptualization (lead); writing – original draft (equal); writing – review and editing (lead).

## CONFLICT OF INTEREST STATEMENT

The authors confirm that there are no conflicts of interest.

## Data Availability

The data presented in this study are available on request from the corresponding author.
